# That H9N2 avian influenza viruses circulating in different regions gather in the same live-poultry market poses a potential threat to public health

**DOI:** 10.3389/fmicb.2023.1128286

**Published:** 2023-02-16

**Authors:** Tengfei Liu, Shumin Xie, Zhiyi Yang, Aimin Zha, Yuting Shi, Lingyu Xu, Junhong Chen, Wenbao Qi, Ming Liao, Weixin Jia

**Affiliations:** ^1^National Avian Influenza Para-Reference Laboratory, College of Veterinary Medicine, South China Agricultural University, Guangzhou, China; ^2^Key Laboratory of Zoonosis, Key Laboratory of Animal Vaccine Development, Ministry of Agriculture and Rural Affairs, Guangzhou, China; ^3^Key Laboratory of Zoonoses Prevention and Control of Guangdong Province, Guangzhou, China

**Keywords:** H9N2, avian influenza virus, live-poultry trade, concentration, spread, potential threat

## Abstract

H9N2 avian influenza viruses are endemic and persistent in China, but those that are prevalent in different provinces are also causes of wide epidemics, related to the spread of wild birds and the cross-regional trade in live poultry. For the past 4 years, beginning in 2018, we have sampled a live-poultry market in Foshan, Guangdong, in this ongoing study. In addition to the prevalence of H9N2 avian influenza viruses in China during this period, we identified isolates from the same market belonging to clade A and clade B, which diverged in 2012–2013, and clade C, which diverged in 2014–2016, respectively. An analysis of population dynamics revealed that, after a critical divergence period from 2014 to 2016, the genetic diversity of H9N2 viruses peaked in 2017. Our spatiotemporal dynamics analysis found that clade A, B, and C, which maintain high rates of evolution, have different prevalence ranges and transmission paths. Clades A and B were mainly prevalent in East China in the early stage, and then spread to Southern China, becoming epidemic with clade C. Strains from different regions converge at the same live-poultry market to communicate, which may be one reasons the H9N2 viruses are difficult to eradicate and increasingly dominant throughout China. Selection pressure and molecular analysis have demonstrated that single amino acid polymorphisms at key receptor binding sites 156, 160, and 190 under positive selection pressure, suggesting that H9N2 viruses are undergoing mutations to adapt to new hosts. Live-poultry markets are important because people who visit them have frequent contact with poultry, H9N2 viruses from different regions converge at these markets and spread through contact between live birds and humans, generating increased risks of human exposure to these viruses and threatening public health safety. Thus, it is important to reducing the cross-regional trade of live poultry and strengthening the monitoring of avian influenza viruses in live-poultry markets to reduce the spread of avian influenza viruses.

## Introduction

The H9N2 viruses are endemic in much of Asia, the Middle East and parts of Africa, causing outbreaks in chickens, quails and other small poultry species, some of which have been transmitted to humans (No Authors Listed, 2014; Peacock et al., 2019; Carnaccini and Perez, 2020; Um et al., 2021). Recently, several cases of human infection with H9N2 viruses have been identified in China (He et al., 2016, 2020; Jie et al., 2018; Zhang et al., 2022), as well as the recombination of swine influenza viruses and H9N2 viruses found in pigs (Sun et al., 2022). Novel avian-derived H7N9 and H10N8 viruses have also infected humans after acquiring H9N2 gene fragments (Ye et al., 2016), which indicates that H9N2 viruses have undergone extensive recombination and continue to circulate in different hosts worldwide, crossing the species barrier and infecting humans directly from poultry, with the potential to cause future outbreaks in poultry and humans (Dong et al., 2011a; Huang et al., 2015; Heidari et al., 2016). As previously stated, live-poultry markets brings people into frequent contact with birds, increasing the risk of human exposure to avian influenza viruses (AIVs) (Huang et al., 2013; Heidari et al., 2016; Li et al., 2016; De Marco et al., 2021; Jin et al., 2022). Therefore, the risk of zoonotic diseases caused by the H9N2 AIVs is increasing and becoming more concerning.

In the early 1990s, the H9N2 began to be isolated sporadically throughout China (Liu H. et al., 2003; Liu J. et al., 2003). With a massive outbreak of an H9N2 epidemic in China from the fall to the winter of 1998, these viruses have became widespread in China (Zhang et al., 2009). Since 2010, there has been a further explosive increase in H9N2 AIVs isolated in China but spreading throughout all regions and provinces, which mainly located in H9.2a and H9.2b (Zhuang et al., 2019). Previous studies divided H9N2 into 16 branches based on the maximum-likelihood (ML) tree of the HA gene, with clade 15 replacing the others as the only dominant branch (Li et al., 2017). In 2012–2013, the H9N2 AIVs in China began to differentiate into three major branches of clades (1–3), and in 2015–2017, clade 1 continued to differentiate into five sub-branches of clades 1.1–1.5, with multiple H9N2 clusters beginning to become co-endemic (Bi et al., 2020). This suggests that a continuing and rapid evolution of the H9N2 viruses is being experienced in China.

The expansion of the avian influenza virus is a complex phenomenon determined by a combination of interactions between host ecology, environmental variables and viral characteristics (Fusaro et al., 2019). Among these are several essential factors of the environmental variable including wild-bird migration, live-bird trade, and climate. One study found that AIVs may be dispersed across several continents by long-distance migratory birds and expanded by different geographic and evolutionary pathways through poultry trade between neighboring countries (Yamaji et al., 2020), and reassortment with local viruses and co-prevalence (Poen et al., 2019; Pohlmann et al., 2019). H9N2 evolved into multiple diverse lineages in China, and at the early stage, there were differences in the strains prevalent in each province, but different branches of the virus have consistently been found in the same location after a period of migration, suggesting that wild-bird migrations and the live-poultry trade are facilitating the spread of the viruses over areas (Jin et al., 2014). These results imply that an investigation of the evolutionary dynamics and transmission routes of H9N2 are critically necessary. Therefore, we have been monitoring the prevalence of H9N2 virus in the same live-poultry market for 4 years and on this basis, we have performed an advanced study on the evolutionary dynamics and transmission routes of H9N2 viruses in recent years to explore the role of live-poultry markets in the spread and evolution of H9N2 in China. Our aim is to provide knowledge for producing guidelines for the prevention and control of H9N2 viruses.

## Materials and methods

### Sample and viruses isolated

A total of 1,270 oropharyngeal and cloacal swabs were collected from chickens, ducks, geese and pigeons in a live-poultry market between 2018 and 2021. Each sample was placed in 1 mL of cold phosphate-buffered saline (PBS) containing penicillin (5,000 U/mL) and streptomycin (5,000 U/mL). After mixing and centrifugation at 10,000 × *g*/min for 5 min, 0.2 mL of supernatant was used to inoculate 9-day-old specific-pathogen-free chicken embryos *via* the allantoic cavity, followed by incubation at 37°C for 48–72 h. We then harvested the allantoic fluid, and a total of 29 viruses were isolated (Supplementary Table 3).

### Sequencing

RNA was extracted from the harvested virus suspensions using the RNeasy Mini Kit (Qiagen, Germany), and the entire full genome sequence was amplified using two-step RT-PCR and the universal primers reported by Hoffmann et al. (2001). The ex Taq premixed enzyme used for the amplification was purchased from the TaKaRa reagent company.^1^ PCR products of the HA and NA segments of these viruses were subjected to agarose gel electrophoresis, and the target fragments were recovered using the QiAamp Gel Extraction Kit (Qiagen, Germany)^2^ and sequenced using an ABI3730 DNA Analyzer (Shenggong Bioengineering Co., Ltd., Shanghai, China). Data were merged and assembled using the Lasergene software^3^ based on the National Center for Biotechnology Information (NCBI) virus database^4^ (accessed on 12 June 2022).

### Maximum likelihood phylogenies of the H9N2 AIVs

The MAFFT version 7.058 was used to align each of the HA and NA gene segments and eliminate those sequences with less than 95% of the expected segment length. Duplicate sequences in the gene fragment were removed using PhyloSuite. We performed the phylogenetic analysis three times using the ML method in IQ-TREE under the GTR + F + G4 model with 1,000 bootstrap replications. A high-quality visualization of the phylogenetic data was performed using the Interactive Tree of Life (iTOL).

### Bayesian maximum clade credibility (MCC) phylogeny and evolutionary dynamics analysis of the H9N2

Based on the phylogenetic topologies obtained and their bootstrap values, we selected several representative reference sequences and formed five smaller datasets. TempEst (version 1.5.1) was used to analyze the *R*^2^ values and correlation coefficient of the temporal signals and the best-fit model in the selected sequences. To estimate the nucleotide substitution rates of HA and NA segments, we used the Bayesian Markov chain Monte Carlo (MCMC) method provided in the Bayesian Evolutionary Analysis Sampling Trees (BEAST) (v1.10.4c) and a relaxed molecular clock model with uncorrelated log-normally distributed rates and a coalescent Bayesian skyline plot. We set the chain lengths to 500 million iterations and performed sampling at every 5,000 steps to obtain an effective sample size (ESS) ≥200, and convergence was assessed using Tracer (v1.7.1). Time-scaled summary of MCC trees with 10% for the post-burn-in posterior were created using TreeAnnotator (v1.10.4), and visualized with FigTree (v1.4.4). The BEAST package was used for the calculation of the tMRCAs of each branch.

### Reference sequence of phylogeographic analysis

The H9N2 viruses that we isolated in the live-poultry market were distributed in three independent branches; therefore, we performed a spatiotemporal analysis of clades A, B, and C, respectively. In order to reduce the potential sampling biases, we randomly subsampled the database in a stratified manner to create a more equitable spatio-temporal distribution of the HA genome sequences of three branches of viruses. To be precise, sequences in each branch were clustered using the CD-HIT program (Huang et al., 2010), and identical sequences within the same time and region were removed. The discrete sampling locations of the clade A, B, and C viruses in this study include Guangdong, Yunnan, Jiangxi, Shandong, Shanghai, Fujian, Jiangsu, Hunan, Henan, Hebei, Hubei, Xinjiang, Ningxia, Chongqing, Guizhou, Guangxi, Sichuan, Shanxi, Beijing, Tianjin, Heilongjiang, and Anhui in China, there are also viruses from Vietnam in clade C. Detailed information regarding the subsampled HA gene sequences of the clade A, B, and C H9N2 subtype viruses used in this study is provide in the Supplementary Table 1.

Time-measured phylogenies were inferred using the Bayesian discrete phylogeographic approach implemented in the BEAST package (v1.10.4). We first performed a regression of root-to-tip genetic distances on the ML tree against exact sampling dates using the TempEst v1.5.3 (Rambaut et al., 2016), which showed a strong temporal signal. Then, we used an uncorrelated lognormal (UCLN) relaxed molecular clock model. In addition, a Bayesian stochastic search variable selection (BSSVS) model with asymmetric substitution was used. For each independent dataset, multiple runs of the MCMC method were combined using LogCombiner (v1.10.4), utilizing 5,000,000,000 total steps for each set, with sampling every 500,000 steps. Subsequently, we used SpreaD3 v0.9.7.1 to develop interactive visualizations of the dispersal process through time and to compute a Bayes factors (BFs) test to assess the support for significant individual transitions between distinct geographic locations (Bielejec et al., 2016). The BF values >100 indicated robust statistical support, 30 < BF values ≤100 indicated very strong statistical support, 10 < BF values ≤30 indicated strong statistical support, 3 < BF values ≤10 indicated substantial statistical support and BF values <3 indicated poor statistical support (Lemey et al., 2009). We used QGIS Version 3.28 to create plots showing the results of the BF tests.^5^

### Selective pressure and receptor binding key site analysis

The selective pressure for each gene segments in clades A, B, and C was determined on the Datamonkey online version of HyPhy package.^6^ The more appropriate number of sequences (*n* ≥ 200) we selected were depend on a study of Ji-Ming et al. (2022). All the sequences in every branch were analyzed by ratio estimation of non-synonymous (dN) to synonymous (dS) substitutions (ω = dN/dS) on a codon-by-codon basis, and ω < 1 indicates negative or purifying selective pressure; ω = 1 implies neutral evolution; and ω > 1 shows positive selection. BioEdit and MEGA 6.0 (Tamura et al., 2013) were used for the analysis of the sequence format conversion and key amino acid changes, and MegAlign was used to analyze the sequence homologies.^7^ We used Weblogo to analyze the conservation of amino acids.^8^ The prediction of potential N-glycosylation sites was performed using the NetNGlyc server 1.0.^9^

## Results

### Prevalence of H9N2 AIVs in China

**FIGURE 1:**
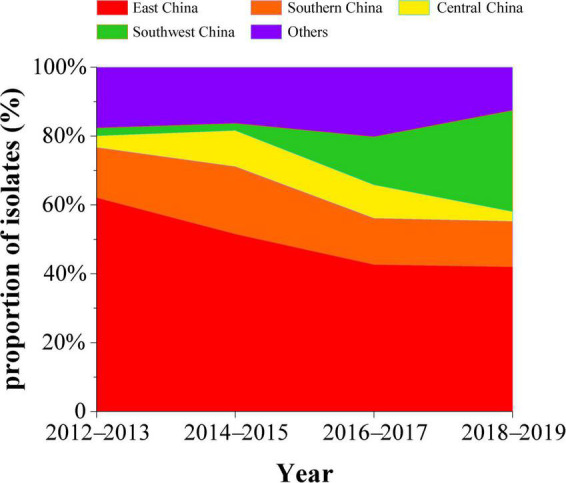
Percentage of isolated H9N2 subtype avian influenza viruses by region in China, 2012–2019 (red, East China; orange, South China; yellow, Central China; green, Southwest China; purple, Other regions).

To explore the prevalence of H9N2 in various regions of China, we counted the percentage of H9N2 avian influenza viruses isolated every year in each region. We found that the number of field isolates of H9N2 in East China accounted for 62.1% of the total isolates in China in 2012–2013. Then this percentage began to decline, with isolates dropping to about 42% in 2018–2019. The isolate percentage of H9N2 AIVs in South China increased to 19.6% in 2014–2015, and then decreased and remained at 13%. The percentage in Southwest China increased from 2% in 2012–2013 to 29.3% in 2018–2019 (Figure 1). The decrease in the percentage of isolates in Guangdong is most likely related to live-poultry markets implementing the “1110 system” of “cleaning and disinfection once a day, a comprehensive sweep once a week, off the market once a month and live-poultry retail markets’ zero stock of live birds on the day of the market.”

### Genetic evolutionary analysis

**FIGURE 2:**
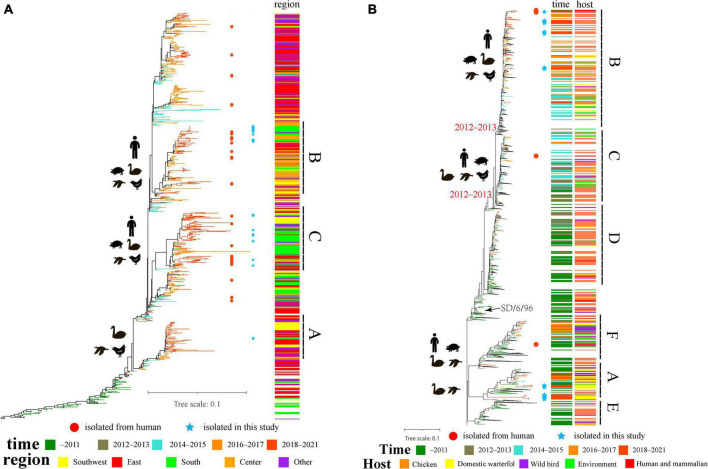
The evolutionary history of H9N2 viruses in China: **(A)** ML tree of the HA gene of H9N2 viruses; **(B)** ML tree of NA gene of H9N2 viruses. Different years, regions and hosts are denoted by different symbols. All branch lengths are scaled according to the numbers of substitutions per site. The tree is rooted using A/chicken/Beijing/1/1994/H9N2. The blue pentagrams represent isolates in this study; the red circles represent human isolates that were isolated in 2016–2021.

We isolated 29 strains of H9N2 avian influenza virus from the same live-poultry market, and the nucleotide identity of HA sequences of all isolates ranged from 92.28 to 100% and the amino acid identity ranged from 93.58 to 100%. The isolated strains were clustered into three groups, I–III (Supplementary Figure 1). Genetic evolutionary analysis revealed three clusters located in branches A, B, and C (the average intergroup distance between branches A and B and C was 0.057 and 0.065, respectively, while the average intergroup distance between B and C was 0.055, which can be considered as forming three independent branches) (Figure 2). Our study found that clade A was first isolated from duck flocks in Zhejiang, and this branch was isolated in live-poultry markets in 2018; however, no further strains of this branch were found in a subsequent surveillance. The clade B branch became endemic in central China in 2016 and then spread to Southern and Southwestern China, and multiple strains of clade B were isolated in live-poultry markets in 2019–2020. Clade C strains were detected in increased numbers in Southern China in 2016 and became epidemic in Guangdong starting in 2018, we continued to isolate strains located in clade C in live poultry markets for 4 years (Figure 2A). We also found that Human-derived H9N2 AIVs does not form a separate branch in the phylogenetic evolutionary tree, and multiple H9N2-induced human infection strains occurring across the country in 2019–2021 are located in clades B and C. According to the chronological order of the prevalence of the strains located in these branches in various regions, the H9N2 AIVs prevalent in Guangdong in recent years may be spread mainly by East China, while the H9N2 viruses prevalent in the Foshan live-poultry market are likely to be spread by strains prevalent in other regions that conduct live-poultry trade.

Genetic evolutionary analysis revealed that the evolutionary tree of the NA gene of the H9N2 viruses, which used to be endemic in China, diverged in 2012–2013 and evolved into three major branches, clade B, clade C and clade D (from A/Chicken/SD/6/96). Among them, clade D strains have rarely been isolated since 2017, while clades B and C are widespread in China, with a wide range of host adaptations including poultry, mammals, and even multiple cases of human-derived infections (Figure 2B). We also isolated many strains located in clade A (belonging to branch A/duck/HK/Y439/97), which has been detected in low numbers in China and is present mainly in wild birds and waterfowl. The nucleotide identity of the NA genes of our isolated strains ranged from 81.49 to 100% and amino acid identity from 85.11 to 100% in the clade A and clade B branches, respectively. The 12 isolates located in clade A were all derived from ducks and geese and did not have neck deletions of the NA protein, consistent with the Y439 branch strain being prevalent mainly in waterfowl. The 16 isolates located in clade B were isolated from chickens, with only one from geese, consistent with the prevalence of Shandong/6/96 in poultry and with the finding that the absence of the NA protein neck enhances the adaptation of the virus from wild birds to poultry.

### Bayesian maximum clade credibility (MCC) phylogeny and evolutionary dynamics analysis

**FIGURE 3:**
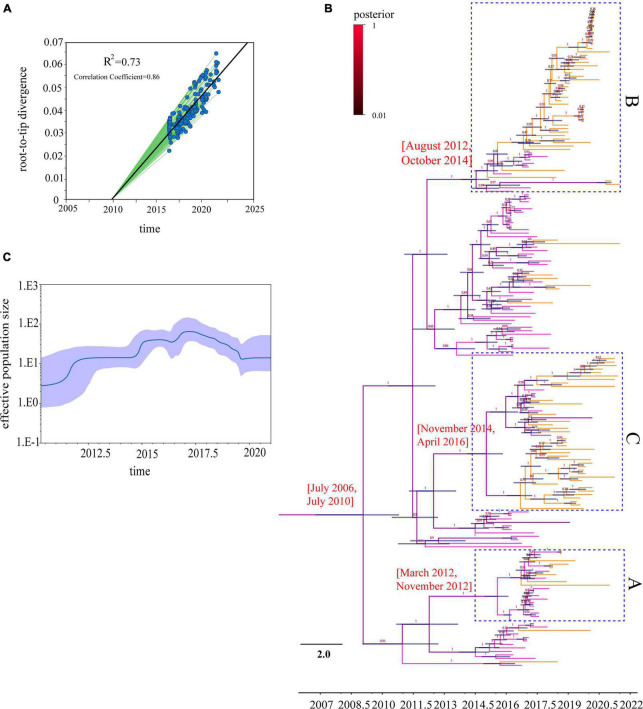
Time-scaled evolution of HA gene of H9N2 viruses in 2016–2021: **(A)** Analysis of root-to-tip divergence against sampling date for HA gene segment, *R*^2^ = 0.73; **(B)** an MCC tree of the HA sequence of H9N2 viruses sampled in China (*n* = 189) in 2016–2021 is shown. H9N2 viruses from different periods are denoted by different colors (pink represents the 2016–2017 H9N2 strains, orange the 2018–2021 strains, and the posterior probabilities of each branch are shown on a scale, with red representing the maximum posterior probability). Shaded bars represent the 95% highest probability distribution for the age of each node; **(C)** a GMRF Bayesian skyline analysis of the HA gene of H9N2 viruses in China in 2016–2021 to display changes in the effective population size over time. The solid red line indicates the median value, and the shaded blue area represents the 95% highest posterior density of genetic diversity estimates.

To analyze the evolutionary relationships of clades A, B, and C with the Chinese H9N2 as a whole, we reconstructed the evolutionary dynamics and estimated the divergence times of the three branches. Root-tip regression analysis of the structure revealed a clock-like structure of the HA gene of the H9N2 subtype of avian influenza virus prevalent in China from 2016 to 2021 (*n* = 189, correlation coefficient = 0.86; *R2* = 0.73) (Figure 3A). Our analysis found that the population diversity of the H9N2 subtype prevalent in China was increasing from 2016 to 2017 and peaked in 2017 (Figure 3B), mainly due to the clade 1 branch beginning to diverge into multiple sub-branches during this period. Genetic diversity decreased significantly starting in 2018, likely due to the dominant branch being dominant, corresponding to the widespread clade C branch prevalence. Previous studies have shown that the evolution rate of the H9N2 virus has increased in recent years (Jin et al., 2020; Wang et al., 2021), which implies an increasing rate of mutation. We estimated the overall average evolution rate of HA genes of H9N2 viruses in China from 2016 to 2021 to be 4.538 × 10^–3^ substitutions/site/year (95% HPD, 4.00 × 10^–3^ to 5.12 × 10^–3^ substitutions/site/year) and the estimated time of tMRCA to be September 2008 (95% HPD, July 2006–2010). Meanwhile, we calculated the evolutionary rates of the three different branches where the HA genes of the isolates were located separately and found that the evolutionary rate of clade A is 5.56 × 10^–3^ substitutions/site/year (95% HPD, 4.50 × 10^–3^ to 6.64 × 10^–3^ substitutions/site/year) and the estimated time of tMRCA to be July 2012 (95% HPD, March 2012–November 2012). The evolutionary rate of clade B is 5.32 × 10^–3^ substitutions/site/year (95% HPD, 4.44 × 10^–3^ to 6.24 × 10^–3^ substitutions/site/year), and the estimated time of tMRCA is March 2012 (95% HPD, April 2009–February 2014). The evolutionary rate of clade C is 5.14 × 10^–3^ substitutions/site/year (95% HPD, 4.16 × 10^–3^ to 6.10 × 10^–3^ substitutions/site/year), and the estimated time of tMRCA is August 2015 (95% HPD, November 2014–April 2016) (Table 1 and Figure 3C). Our study shows that 2012–2013 and 2014–2016 are two critical periods for the divergence of the H9N2 subtype AIV in China, and during this period, the evolutionary rate of HA genes was maintained at a high level. The difference between the evolutionary rate of the HA gene as a whole and the evolutionary rate calculated for each branch individually suggests that the branches of H9N2 are not evolving at the same rate; some branches are spreading widely and have a higher probability of communicating with other strains, while the use of vaccines in widespread areas may also lead to a higher probability of the virus to mutate to escape immunity.

**TABLE 1:** Evolutionary rates of HA and NA genes from 2013 to 2021.

Substitution rates (subs/site/year)					
Clade	Period	ESS	Mean rate	Upper (95% HPD)	Lower (95% HPD)
Clade A	2013–2018	1,234	5.56E-3	4.50E-3	6.64E-3
Clade B	2015–2021	324	5.25E-3	4.46E-3	5.94E-3
Clade C	2016–2021	564	5.14E-3	4.16E-3	6.10E-3
HA (overall)	2016–2021	1,680	4.54E-3	4.00E-3	5.12E-3
NA (overall)	2016–2021	1,680	5.50E-3	4.84E-3	6.20E-3

### Phylogeographic analysis

**FIGURE 4:**
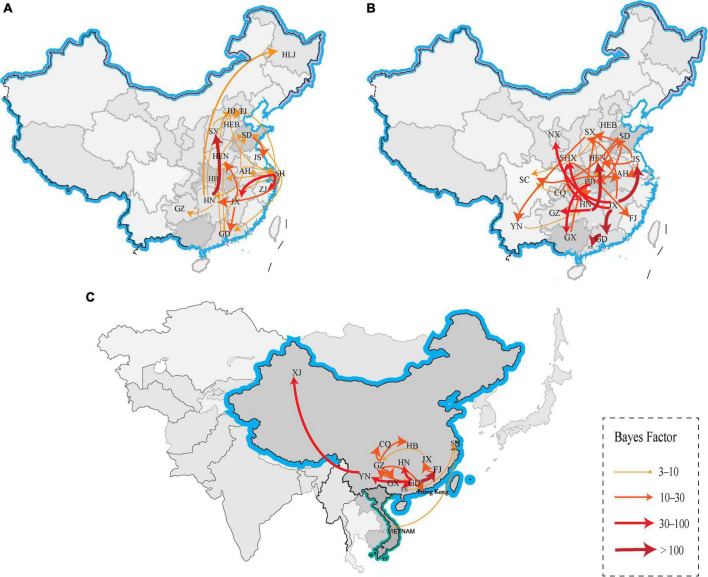
Spatiotemporal dissemination of clade A, B and C H9N2 viruses from 2012 to 2021, which was determined by the Bayesian phylogeographic inference of the HA gene sequences. **(A)** The transmission route of clade A, **(B)** the transmission route of clade B, and **(C)** the transmission route of clade C. Curves show the among-province virus lineage transitions statistically supported with BF >3 for H9N2 viruses. Curve widths and colors represent the corresponding statistical support (BF value) for each transition rate. The abbreviations in the map represent GD, Guangdong; YN, Yunnan; JX, Jiangxi; SD, Shandong; SH, Shanghai; FJ, Fujian; JS, Jiangsu, HN, Hunan; HEN, Henan; HEB, Hebei; HB, Hubei; XJ, Xinjiang; NX, Ningxia; CQ, Chongqing; GZ, Guizhou; GX, Guangxi; SC, Sichuan; SX, Shanxi; BJ, Beijing; TJ, Tianjin; HLJ, Heilongjiang; AH, Anhui; and FS, Foshan.

We reconstructed the spatial dispersal networks of the three branches based on the HA genes and found that the clade A viruses spread mainly among Chinese provinces, with Shanghai → Hunan (*BF* = 12), Shanghai → Jiangxi (*BF* = 33), Shanghai → Zhejiang (*BF* = 47), Hunan → Shanxi (*BF* = 207) and Jiangxi → Guangdong (*BF* = 22) as the main routes (Table 2 and Figure 4A). We also found that Jiangxi → Guangdong (migration rate = 1.54) had a higher migration rate, and that Shanghai, Jiangxi, Jiangsu, and Hunan are the main prevalent geographic locations of this branch and closely connected with other regions (Supplementary Table 2). The clade B viruses spread mainly in the trajectories of Jiangxi → Jiangsu (*BF* = 3,386), Jiangxi → Guangdong (*BF* = 849), Jiangxi → Ningxia (*BF* = 48), Jiangxi → Shanxi (*BF* = 52), and Hunan → Henan (*BF* = 112) and Hunan → Guizhou (*BF* = 62) (Table 2 and Figure 4B). The strain we detected in Foshan was probably transmitted from within Guangdong Province (*BF* = 849), and it can be shown that Jiangxi and Hunan are the main endemic areas of the clade B viruses, with strong links to other regions. The clade C viruses spread mainly with the trajectories Guangdong → Fujian (*BF* = 161), Guangdong → Guizhou (*BF* = 23), Guangdong → Hunan (*BF* = 39), Guangdong → Yunnan (*BF* = 58), Fujian → Jiangxi (*BF* = 15) and Yunnan → Xinjiang (*BF* = 65) (Table 2 and Figure 4C), and the results show that Guangdong is the epidemiological center of this branch and is closely linked to several regions. The strains isolated from our sampling site in Foshan are also very likely to be locally transmitted in Guangdong. Our findings suggest that different branches of the strain are prevalent in South and East China, respectively, with the clade A and clade B branches predominant in Shanghai, Jiangxi, and Hunan, and the clade C branch predominant in Guangdong, which is consistent with the need for different H9 subtype vaccines in each region of China and may also be the primary reason H9N2 AIV has been difficult to control in China.

**TABLE 2:** Bayes factor and posterior-probability support for all location transitions.

Branch	From	To	Bayes factor	Posterior-probability
Clade A	Shandong	Jiangsu	11.0073866	0.435062771
	Shanghai	Hunan	11.96797603	0.455727141
	Shanghai	Jiangxi	33.058187	0.698144651
	Shanghai	Zhejiang	47.11671903	0.767248084
	Jiangxi	Guangdong	22.2873058	0.609265637
	Hunan	Heilongjiang	16.71513357	0.539051217
	Hunan	Shanxi	207.5239226	0.935562715
	Jiangxi	Henan	10.42408548	0.421730919
Clade B	Jiangsu	Henan	10.01298786	0.341628708
	Shanxi	Fujian	10.17711743	0.345294967
	Jiangsu	Shanxi	10.53142937	0.353071881
	Guangxi	Hubei	11.914291	0.381735363
	Shandong	Sichuan	14.68004257	0.432063104
	Chongqing	Anhui	18.38805695	0.487945784
	Jiangxi	Shanxi	18.77630282	0.493167426
	Jiangsu	Anhui	18.8264427	0.493834018
	Hubei	Shandong	20.15118074	0.51083213
	Shanxi	Changsha	20.83477169	0.519164537
	Shandong	Hubei	23.01486674	0.543939562
	Shanxi	Yunnan	25.10231769	0.565381624
	Shanxi	Hunan	34.77829042	0.643150761
	Hubei	Guangxi	37.85658973	0.662370848
	Jiangxi	Ningxia	47.81643401	0.712476392
	Jiangxi	Shaanxi	52.0920264	0.7296967
	Changsha	Guizhou	62.74804805	0.764803911
	Changsha	Henan	112.0865313	0.85312743
	Jiangxi	Guangdong	569.4778853	0.967225864
	Guangdong	Foshan	849.1457537	0.977780247
	Jiangxi	Jiangsu	3386.359614	0.994333963
Clade C	Guizhou	Chongqing	12.44319747	0.483501833
	Guizhou	Hubei	12.8252143	0.491056549
	Guizhou	Guangxi	14.42239848	0.520386624
	Fujian	Jiangxi	15.75464552	0.54238418
	Guizhou	Hongkong	16.46258092	0.553271859
	Guangdong	Guizhou	23.84129334	0.642039773
	Guangdong	Foshan	38.32310441	0.742473059
	Guangdong	Hunan	39.62433504	0.748805688
	Guangdong	Yunnan	58.48008009	0.814798356
	Yunnan	Xinjiang	64.90676416	0.830018887
	Guangdong	Fujian	161.3713982	0.923897345

### Selective pressure and receptor binding key site analysis

**FIGURE 5:**
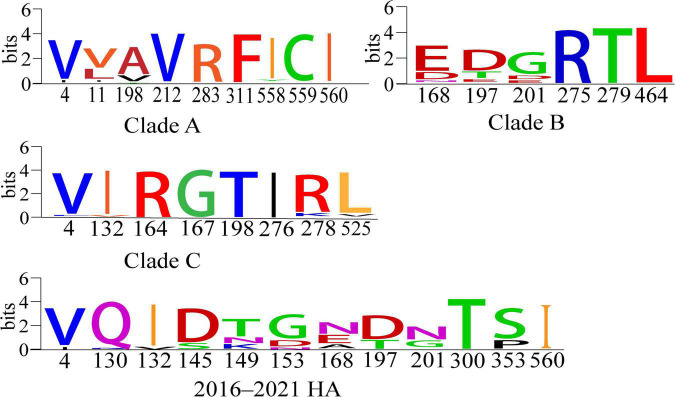
Conserved amino acid analysis of positive-selection pressure sites for clades A, B and C and 2016–2021 HA genes of H9N2 viruses (Crooks et al., 2004). Each logo consists of stacks of symbols, one stack for each position in the sequence. The overall height of the stack indicates the sequence conservation at that position, while the height of the symbols within the stack indicates the relative frequency of each amino or nucleic acid at that position.

Positive selection leads to an increase in the number of genetic variants, allowing the preservation of mutations that favor viral adaptation and survival, while negative selection purifies unfavorable mutations and tends to conserve genes (Yang and Nielsen, 2002; Kosiol et al., 2006). We used the Branch model aBSREL (Smith et al., 2015) to analyze the HA genes clades A, B, and C for selection pressure and found that they were all under purifying selection pressure (Clade A, 0.102; B, 0.117; C, 0.161, *p* = 1). Then, when we analyzed with the site model MEME (Murrell et al., 2012), FUBAR (Murrell et al., 2013), SLAC (Kosakovsky and Frost, 2005), we identified a total of 12 positively selected pressure sites for the HA gene of H9N2 AIVs prevalent in China from 2016 to 2021, and clades A, B, and C have 9, 6, and 8 positive selection pressure points, respectively (Table 3 and Figure 5). Mutations in amino acids at these sites facilitate the adaptation of H9N2 avian influenza viruses to unfavorable environments.

**TABLE 3:** Positive selection pressure sites for the HA gene of H9N2 viruses.

Mode				
Clade	MEME (*P* > 0.9)	FUBAR (*P* > 0.9)	SLAC (*P* > 0.9)	Consensus site
Clade A	558,560,198,559,311,11,212,283	198,11,311,283,4	198,11,283	11,198,283
Clade B	197,275,279,464,168	168	168,201	168
Clade C	278,167,525,198,276,164,4,132	525	164,525,278	525
2016-2021 (HA)	4,130,145,149,153,197,300,353,560	4,201	4,132,168,201,353	4

**FIGURE 6:**
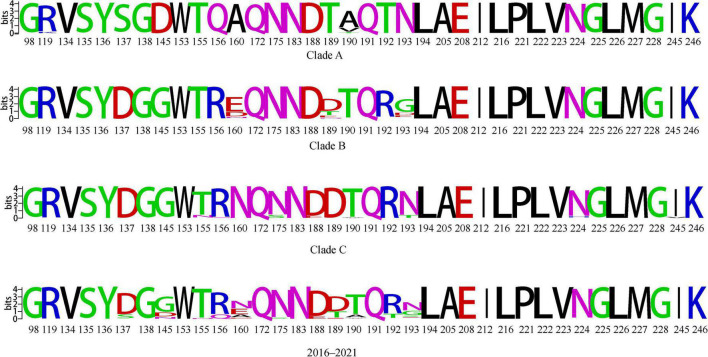
Conserved amino acid analysis of key receptor-binding sites of clades A, B and C and 2016–2021 HA genes of H9N2 viruses. Each logo consists of stacks of symbols, one stack for each position in the sequence. The overall height of the stack indicates the sequence conservation at that position, while the height of symbols within the stack indicates the relative frequency of each amino or nucleic acid at that position.

Receptor-binding preference is important for influenza virus replication and transmission (Herfst et al., 2012), and mutations in multiple amino acid sites located in the HA protein affect the receptor-binding propensity of H9N2 AIVs. We found that 13 of the receptor-binding sites for the HA proteins of H9N2 viruses isolated in China from 2016 to 2021 were non-conserved (Figure 6). Two of the sites are not conserved in clade A; three sites are not conserved in clade B, and up to eight sites are not conserved in clade C. Compared to the other two branches, clade A had the fewest number of mutations at key sites. At the same time, we found that strains located in clade B and clade C have mutations at 145 from D to G, 190 from A to T/V, 160 from A to E/D/N, and 156 from Q to R. These sites are conserved in clade A strains, and mutations increase the binding properties of the strains to human receptors. These findings are consistent with clade B and clade C being responsible for human infection and having a greater prevalence.

We found amino acid polymorphisms in several sites of HA proteins located in different branches (Supplementary Table 4), including key sites 145, 155, 160, and 190 that affect receptor binding, and the presence of polymorphisms at these sites may contribute to the differential ability of the corresponding strains to infect humans. Combined with the selective pressure analysis, we found that the 190 point of clade A, the 160 point of clade B, and the 156 and 190 key receptor-binding sites of clade C are under positive selection. It is possible that the wide- spread use of vaccines for prevention of H9N2 AIVs has resulted in several antigenic sites under positive selection pressure, at the same time, some sites are also receptor binding key sites and their mutations facilitate viral adaptation to new hosts (Supplementary Table 5 and Figure 6).

The cleavage sites of clade B and clade C include the forms PTRSSR↓GLF, PSRYSR↓GLF, RSRYSR↓GLFPSKSSR↓GLF, PSRYSR↓GLF and PSRSGR↓GLF, in addition to the PSRSSR↓GLF possessed by clade A (Supplementary Table 5). Mutations in receptor-binding sites and the diversity of cleavage sites may lead to variability in the transmission efficiency of different branches of the strains across multiple hosts, which in turn affects the prevalence of this branch. we found that the HA proteins of clades A, B, and C were not only missing 215–218 potential glycosylation sites but also a new 313–315 glycosylation site near the cleavage site compared to previous reports (Li et al., 2005; Dong et al., 2011b; Liu et al., 2016). Increased glycosylation sites may significantly increase the ability of viruses to infect mammalian cells and avian species (Peng et al., 2019; Yang et al., 2021), and also increase the ability of viruses to escape immunity to vaccines (Tate et al., 2014).

It has been shown that deletion of the NA protein neck enhances the adaptation of the viruses from wild birds to poultry and also enhances the replication of the viruses in mammals. Phylogenetic analysis of the NA gene showed multiple cases of human infection in both clade B and clade C with neck deletion, but no human infection has been reported in clade A. Meanwhile, our analysis of key sites revealed no mutations at points 274 and 294 in clade A and clade B, maintaining susceptibility to oseltamivir and neuraminidase inhibitors.

### Molecular characterization of isolates from the live-poultry market

We analyzed the HA genes of viruses isolated from live-poultry markets and found that all isolates had the PSRSSR↓GLF cleavage site, consistent with a molecular signature of low pathogenicity. The Q226L and H183N mutations were present in all isolates; the I155T mutation was present in 28 isolates; one isolate had the I155N mutation; and 27 isolates had the A190T mutation. A new glycosylation site, 313–315, was present near the cleavage site, indicating that all these isolates are potential risks for human infection. We found that all 12 isolates located in clade A had ITE at sites 59–61 of the NA protein, and 17 isolates located in clade B had missing sites 59–61, indicating that the live-poultry market has a mixture of NA protein neck-deficient and non-deficient strains of H9N2 AIVs, thereby giving the viruses a greater chance of co-transmission and circulation in wild waterfowl and poultry.

## Discussion

Since the end of the 20th century, there has been a widespread epidemic of H9N2 in China; mainly, parasitic G1 strains of quail are prevalent in Southern China, and BJ/94 and F/98 strains are prevalent predominantly in flocks in Northern and Eastern China (Sun et al., 2010). Since 2010, H9N2 viruses isolated from vaccinated chickens have caused widespread disease in China (Xia et al., 2017), and since 2011, the prevalent H9N2 strains have belonged mainly to the h9.4.2.5 or h9.4.2.6 lineages (Jiang et al., 2012). Subsequently, with the extensive use of vaccines against H9 AIVs in China, the isolation rate of the h9.4.2.6 lineage gradually decreased, and the h9.4.2.5 lineage became the main prevalent branch. Studies found that the antisera titers of strains from different sub-branches of the h9.4.2.5 lineage were different (Liu et al., 2016; Xu et al., 2018), which suggests that further divergence is occurring in the h9.4.2.5 lineage. Li et al. (2017) had divided H9N2 viruses isolated in China in 1994–2013 into 15 branches, and the absolutely dominant branch of the Chinese epidemic was clade 15, which has the strain A/CK/SD/JN/1999/H9N2 as its representative. The use of vaccines may accelerate virus evolution; a study has shown that, during 2010–2013, H9N2 AIVs underwent antigenic drift from commercial vaccines, causing outbreaks nationwide with the emergence of new antigenic clusters (Wei et al., 2016). Therefore, a more precise method of dividing clusters to study the characteristics of currently prevalent strains is needed. We found that the field strains in this study are located in three sub-branches of clade 15, also belonging to h9.4.2.5, and the distance between groups we calculated for the three branches have shown that they are independent. These results suggest that, although our isolates from the live-poultry market still belong to the dominant branch, branch 15 has diverged in its continued evolution, and the strains we isolated not only have the potential to escape immunity from current commercial vaccines, but there may also be antigenic differences between the three branches. The continued evolution of H9N2 in China and the failure of several classical vaccines to provide effective prevention and control of the current epidemic of H9N2 suggest the need for permanent and continuous monitoring of H9N2 viruses.

The antigenic drift of avian influenza viruses occurs rapidly, and single-site mutation in the HA and NA proteins could alter the structure of the viral surface proteins, resulting in the production of antigenic variants. Mutations of single sites of viruses could be reflected by nucleotide substitution rates. In recent years, nucleotide substitution rates of HA and NA genes have shown an upward trend (Jin et al., 2020). We found that the HA gene of H9N2 maintained a high evolutionary rate starting in 2013, suggesting that the mutation rate of H9N2 viruses has been sustained at a relatively high level and that the antigenic variability of H9N2 viruses is rapid. Calculating the most-recent common ancestor allows us to estimate the divergence time of a branch. We found that 2012–2013 was the period when clades A and B emerged, and 2015–2017 is when clade C appeared, which indicates that 2012–2013 and 2014–2016 were two important periods for the divergence of H9N2 viruses. The population dynamics of H9N2 reflect the dynamics of the genetic diversity of viral populations over time. We found that multiple branches emerged from 2015 to 2016, and the population diversity of H9N2 peaked in 2017, then began to decline and stabilize. We speculate that this is related to the widespread existence of clades B and C that started to dominate in China after 2017. This suggests to us that we should focus on strengthening the monitoring of branches B and C.

Available studies have demonstrated that the transport of live poultry has more significant effects on the spread of the virus than climatic effects and has been shown to facilitate the spread of AIVs epidemics (Li et al., 2018; Chen et al., 2020). After the introduction of H9N2 AIVs in China, South China was identified as the center of the epidemic. However, since the implementation of the new live-poultry trading policy in Guangdong Province, the proportion of H9N2 subtype AIVs isolated in Southern China has decreased. After 2013, the viruses isolated from several provinces were most similar to the strains from Eastern China (Zhang et al., 2019; Gao et al., 2021; Guo et al., 2021; Hu et al., 2021; Liu et al., 2022), which is consistent with our analysis that the epidemiological center of H9N2 AIVs has shifted to Eastern China. With our monitoring of the live-poultry market in Foshan, we found that Eastern China was an early epidemic center for clade A and clade B. It is likely that isolates from both these clades were spread from Jiangxi to Guangdong and dispersed into Foshan again within the province. Clade C mainly disseminated into Foshan within Guangdong and transmitted to other provinces. We also found that clades B and C had wide-ranging prevalence and broader host infection. Several isolates from humans were located in the B and C branches, while no human isolates have been reported from A branch. These results imply that clades B and C pose a higher risk to human health and farming than does clade A.

Different strains converge in the same location and are vulnerable to recombination, thereby threatening human health. The H9N2 viruses belong to different branches and are prevalent in East and South China, but we were able to detect strains belonging to these branches in a live-bird market in Guangdong, indicating that live-poultry transport plays an important role in the transmission and recombination of H9N2 AIVs. Our continuous surveillance of the same live-poultry market revealed that the isolated H9N2 AIVs had mutations in multiple receptor-binding key sites and were under positive selection pressure and similar to human isolates. This suggests that H9N2 viruses prevalent in live-poultry markets are already capable of infecting humans, that mutations may also increase their ability to bind to human receptors, and that close contact with live poultry puts humans at increased risk of contracting H9N2 AIVs. Therefore, the continuous monitoring of live-poultry markets and the reduction of cross-regional trade of live poultry are important in order to protect public health and safety.

## Data availability statement

The data presented in this study are deposited in the GISAID’s EpiFlu™. Accession numbers were supported in Supplementary Table 3.

## Author contributions

TL: conceptualization, methodology, software, validation, formal analysis, and writing—original draft preparation and editing. SX: investigation and resources. ZY: investigation. AZ: investigation, resources, data curation, and writing—review and editing. YS: visualization and supervision. WQ and ML: project administration. WJ: project administration and funding acquisition and editing. All authors contributed to the article and approved the submitted version.

## Footnotes

1
https://www.takarabio.com

2
https://www.qiagen.com/us

3
https://www.dnastar.com/software/

4
https://www.ncbi.nlm.nih.gov/

5
https://www.qgis.org/en/site/

6
http://www.datamonkey.org/

7
https://www.dnastar.com/software/lasergene/megalign-pro/

8
http://weblogo.Berkeley.edu/logo.cgi

9
http://www.cbs.dtu.dk/services/NetNGlyc/
