# Supplementary material for: That H9N2 avian influenza viruses circulating in different regions gather in the same live-poultry market poses a potential threat to public health
Source: Front Microbiol. 2023 Feb 16;14:1128286. doi: 10.3389/fmicb.2023.1128286 (PMC9979309; doi:10.3389/fmicb.2023.1128286)
Supplement: Supplementary file 3 [file Table_3.DOCX]

Table S3. H9N2 strains isolated from 2018 to 2021 in the live-poultry market in Foshan, Guangdong.

| Gene | Clade | Name | Isolate_Id | Collect time |
| --- | --- | --- | --- | --- |
| HA | A | A/Chicken/Foshan/F717/2018/H9N2 | [EPI_ISL_14606437](https://platform.epicov.org/epi3/start/EPI_ISL/14606437) | 2018-06-22 |
|  |  | A/Chicken/Foshan/F733/2018/H9N2 | [EPI_ISL_14606940](https://platform.epicov.org/epi3/start/EPI_ISL/14606940) | 2018-06-22 |
|  | B | A/Goose/Foshan/G860/2019/H9N2 | [EPI_ISL_14619758](https://platform.epicov.org/epi3/start/EPI_ISL/14619758) | 2019-12-11 |
|  |  | A/Goose/Foshan/G855/2019/H9N2 | [EPI_ISL_14619442](https://platform.epicov.org/epi3/start/EPI_ISL/14619442) | 2019-12-11 |
|  |  | A/Goose/Foshan/G850/2019/H9N2 | [EPI_ISL_14619023](https://platform.epicov.org/epi3/start/EPI_ISL/14619023) | 2019-12-11 |
|  |  | A/Goose/Foshan/G836/2019/H9N2 | [EPI_ISL_14619021](https://platform.epicov.org/epi3/start/EPI_ISL/14619021) | 2019-12-11 |
|  |  | A/Goose/Foshan/G866/2019/H9N2 | [EPI_ISL_14620172](https://platform.epicov.org/epi3/start/EPI_ISL/14620172) | 2019-12-11 |
|  |  | A/Chicken/Foshan/G533/2019/H9N2 | [EPI_ISL_14619010](https://platform.epicov.org/epi3/start/EPI_ISL/14619010) | 2019-07-08 |
|  |  | A/Chicken/Foshan/G521/2019/H9N2 | [EPI_ISL_14615530](https://platform.epicov.org/epi3/start/EPI_ISL/14615530) | 2019-07-08 |
|  |  | A/Chicken/Foshan/G525/2019/H9N2 | [EPI_ISL_14618980](https://platform.epicov.org/epi3/start/EPI_ISL/14618980) | 2019-07-08 |
|  |  | A/Chicken/Foshan/G524/2019/H9N2 | [EPI_ISL_14618799](https://platform.epicov.org/epi3/start/EPI_ISL/14618799) | 2019-07-08 |
|  |  | A/Chicken/Foshan/G519/2019/H9N2 | [EPI_ISL_14615529](https://platform.epicov.org/epi3/start/EPI_ISL/14615529) | 2019-07-08 |
|  |  | A/Chicken/Foshan/G517/2019/H9N2 | [EPI_ISL_14615528](https://platform.epicov.org/epi3/start/EPI_ISL/14615528) | 2019-07-08 |
|  |  | A/Duck/Foshan/H51/2020/H9N2 | [EPI_ISL_14605231](https://platform.epicov.org/epi3/start/EPI_ISL/14605231) | 2020-01-07 |
|  |  | A/Duck/Foshan/G861/2019/H9N2 | [EPI_ISL_14620107](https://platform.epicov.org/epi3/start/EPI_ISL/14620107) | 2019-12-11 |
|  |  | A/Duck/Foshan/G853/2019/H9N2 | [EPI_ISL_14619088](https://platform.epicov.org/epi3/start/EPI_ISL/14619088) | 2019-12-11 |
|  |  | A/Duck/Foshan/G843/2019/H9N2 | [EPI_ISL_14619022](https://platform.epicov.org/epi3/start/EPI_ISL/14619022) | 2019-12-11 |
|  |  | A/Duck/Foshan/H54/2020/H9N2 | [EPI_ISL_14620176](https://platform.epicov.org/epi3/start/EPI_ISL/14620176) | 2020-01-07 |
|  |  | A/Duck/Foshan/H45/2020/H9N2 | [EPI_ISL_14620175](https://platform.epicov.org/epi3/start/EPI_ISL/14620175) | 2020-01-07 |
|  |  | A/Duck/Foshan/H36/2020/H9N2 | [EPI_ISL_14620173](https://platform.epicov.org/epi3/start/EPI_ISL/14620173) | 2020-01-07 |
|  | C | A/Chicken/Foshan/F1007/2018/H9N2 | [EPI_ISL_14583508](https://platform.epicov.org/epi3/start/EPI_ISL/14583508) | 2018-09-11 |
|  |  | A/Chicken/Foshan/I28/2021/H9N2 | [EPI_ISL_14620234](https://platform.epicov.org/epi3/start/EPI_ISL/14620234) | 2021-01-11 |
|  |  | A/Chicken/Foshan/I27/2021/H9N2 | [EPI_ISL_14620232](https://platform.epicov.org/epi3/start/EPI_ISL/14620232) | 2021-01-11 |
|  |  | A/chicken/Foshan/F1025/2018/H9N2 | [EPI_ISL_356405](https://platform.epicov.org/epi3/start/EPI_ISL/356405) | 2018-09-11 |
|  |  | A/Chicken/Foshan/F998/2018/H9N2 | [EPI_ISL_14615163](https://platform.epicov.org/epi3/start/EPI_ISL/14615163) | 2018-09-11 |
|  |  | A/Chicken/Foshan/G825/2019/H9N2 | [EPI_ISL_14619019](https://platform.epicov.org/epi3/start/EPI_ISL/14619019) | 2019-12-11 |
|  |  | A/Chicken/Foshan/G817/2019/H9N2 | [EPI_ISL_14619017](https://platform.epicov.org/epi3/start/EPI_ISL/14619017) | 2019-12-11 |
|  |  | A/Chicken/Foshan/G403/2019/H9N2 | [EPI_ISL_14615527](https://platform.epicov.org/epi3/start/EPI_ISL/14615527) | 2019-05-09 |
|  |  | A/Chicken/Foshan/G523/2019/H9N2 | [EPI_ISL_14618103](https://platform.epicov.org/epi3/start/EPI_ISL/14618103) | 2019-07-08 |
