# Supplementary material for: That H9N2 avian influenza viruses circulating in different regions gather in the same live-poultry market poses a potential threat to public health
Source: Front Microbiol. 2023 Feb 16;14:1128286. doi: 10.3389/fmicb.2023.1128286 (PMC9979309; doi:10.3389/fmicb.2023.1128286)
Supplement: Supplementary file 4 [file Table_4.DOCX]

Table S4. Single amino acid polymorphism of HA protein in viruses of Clade A, B and C.

| Clade | Position (H3 numbering) | | | | | | | | | | | | | |
| --- | --- | --- | --- | --- | --- | --- | --- | --- | --- | --- | --- | --- | --- | --- |
|  | 11 | 87 | 88 | 90 | 91 | 105 | 115 | 126 | 145 | 149 | 150 | 153 | 163 | 165 |
| A | V | P | L | G | G | N | N | S | S | K | A | D | T | Q |
| B | L | L | S | E | G | H | D | A | D | T | A | G | T | R |
| C | L | P | L | E | E | N | N | A | D | S+N | T+A | G | T+N | Q+R |
|  | 166 | 168 | 171 | 192 | 197 | 198 | 200 | 201 | 217 | 266 | 320 | 353 | 377 | 510 |
| A | N | A | I | H | T | A | T | N | M | H | V | S | D | K |
| B | N | E | T | H | D | T | R | G | I | H | V | P | D | R |
| C | D+N | N | I | N+H | D | T | R | N+T | I | Y | I | S+P | E | K |
