# Supplementary material for: That H9N2 avian influenza viruses circulating in different regions gather in the same live-poultry market poses a potential threat to public health
Source: Front Microbiol. 2023 Feb 16;14:1128286. doi: 10.3389/fmicb.2023.1128286 (PMC9979309; doi:10.3389/fmicb.2023.1128286)
Supplement: Supplementary file 5 [file Table_5.DOCX]

Table S5. Analysis of receptor binding key sites of HA proteins from Clade A, B and C viruses.

| Segment | Position  H3. NO | Position  H9. NO | Clade | | | Mutation | Function |
| --- | --- | --- | --- | --- | --- | --- | --- |
|  |  |  | A | B | C |  |  |
| HA | 119 | 109 | R | R | R | S→R | Increasing bingding to human-like receptor[1-4] |
|  | 145 | 135 | D | G | G | D→G/N |  |
|  | 155 | 145 | T | T | T/N/Y | I→T |  |
|  | 190 | 180 | A/V/T | T | T/V | A→T/V |  |
|  | 226 | 216 | L | L | L | Q→L |  |
|  | 227 | 217 | M | M | M | Q→M |  |
|  | 98 | 88 | G | G | G |  | Forming the base of the RBS[5] |
|  | 136 | 126 | Y | Y | Y |  |  |
|  | 153 | 143 | W | W | W |  |  |
|  | 183 | 173 | N | N | N | H→N |  |
|  | 225 | 215 | G | G | G | G→E | Increasing the efficiency of viral assembly and budding[6] |
|  | 228 | 218 | G | G | G | G→S | Changing the receptor preference[7, 8] |
|  | 205 | 195 | A | A | A | T→A | Increasing binding to human-like receptors[2, 9] |
|  | 208 | 198 | E | E | E | D→E |  |
|  | 216 | 206 | L | L | L | V→L |  |
|  | 246 | 236 | K | K | K | R→K |  |
|  | 245 | 235 | I | I | I/V | V→I |  |
|  | 160 | 150 | A | E/D | N | A→D |  |
|  | 212 | 202 | I | I/V | I/V | T→I |  |
|  | 156 | 146 | Q | R | R/Q | Q→R |  |
|  | 172 | 162 | Q | Q | Q | R→Q |  |
|  | 175 | 165 | N | N | S/N | S→N |  |
|  | 188 | 178 | D | D | D/E | D→Y |  |
|  | Cleavage site |  | PSRSSR↓GLF | PSRSSR↓GLF/ PTRSSR↓GLF/  PSRYSR↓GLF/ RSRYSR↓GLF | PSRSSR↓GLF/ PSKSSR↓GLF/  PSRYSR↓GLF/ PSRSGR↓GLF |  |  |
| Segment | Position  N2. NO |  | Clade A | Clade B | Mutation | |  |
| NA | 62-64 |  | ITE | delete |  |  | 63-65 deletion in NA increases replication in mammals[10]. |
|  | 274 |  | H | H | H→Y | | conferring resistance to oseltamivir[11]. |
|  | 294 |  | N | N | N→S | | confer resistance to neuraminidase inhibitors[12]. |

**References:**

[1]. Yang, F., et al., Molecular characterization and antigenic analysis of reassortant H9N2 subtype avian influenza viruses in Eastern China in 2016. Virus Res, 2021. 306: p. 198577.

[2]. Liu, Y., et al., Variation and Molecular Basis for Enhancement of Receptor Binding of H9N2 Avian Influenza Viruses in China Isolates. Front Microbiol, 2020. 11: p. 602124.

[3]. Zou, S., et al., Molecular characterization and receptor binding specificity of H9N2 avian influenza viruses based on poultry-related environmental surveillance in China between 2013 and 2016. Virology, 2019. 529: p. 135-143.

[4]. Nobusawa, E., et al., Change in receptor-binding specificity of recent human influenza A viruses (H3N2): a single amino acid change in hemagglutinin altered its recognition of sialyloligosaccharides. Virology, 2000. 278(2): p. 587-96.

[5]. Martin, J., et al., Studies of the binding properties of influenza hemagglutinin receptor-site mutants. Virology, 1998. 241(1): p. 101-11.

[6]. Wang, Z., et al., A Single-Amino-Acid Substitution at Position 225 in Hemagglutinin Alters the Transmissibility of Eurasian Avian-Like H1N1 Swine Influenza Virus in Guinea Pigs. J Virol, 2017. 91(21).

[7]. Vines, A., et al., The role of influenza A virus hemagglutinin residues 226 and 228 in receptor specificity and host range restriction. Journal of virology, 1998. 72(9).

[8]. Wan, H. and D.R. Perez, Amino acid 226 in the hemagglutinin of H9N2 influenza viruses determines cell tropism and replication in human airway epithelial cells. J Virol, 2007. 81(10): p. 5181-91.

[9]. Peacock, T.P., et al., Genetic determinants of receptor-binding preference and zoonotic potential of H9N2 avian influenza viruses. J Virol, 2020.

[10]. Li, X., et al., Genetics, receptor binding property, and transmissibility in mammals of naturally isolated H9N2 Avian Influenza viruses. PLoS Pathog, 2014. 10(11): p. e1004508.

[11]. Weinstock, D.M., L.V. Gubareva and G. Zuccotti, Prolonged shedding of multidrug-resistant influenza A virus in an immunocompromised patient. The New England journal of medicine, 2003. 348(9).

[12]. Kiso, M., et al., Resistant influenza A viruses in children treated with oseltamivir: descriptive study. Lancet, 2004. 364(9436): p. 759-65.
