# Supplementary figures and images for: That H9N2 avian influenza viruses circulating in different regions gather in the same live-poultry market poses a potential threat to public health
Source: Front Microbiol. 2023 Feb 16;14:1128286. doi: 10.3389/fmicb.2023.1128286 (PMC9979309; doi:10.3389/fmicb.2023.1128286)

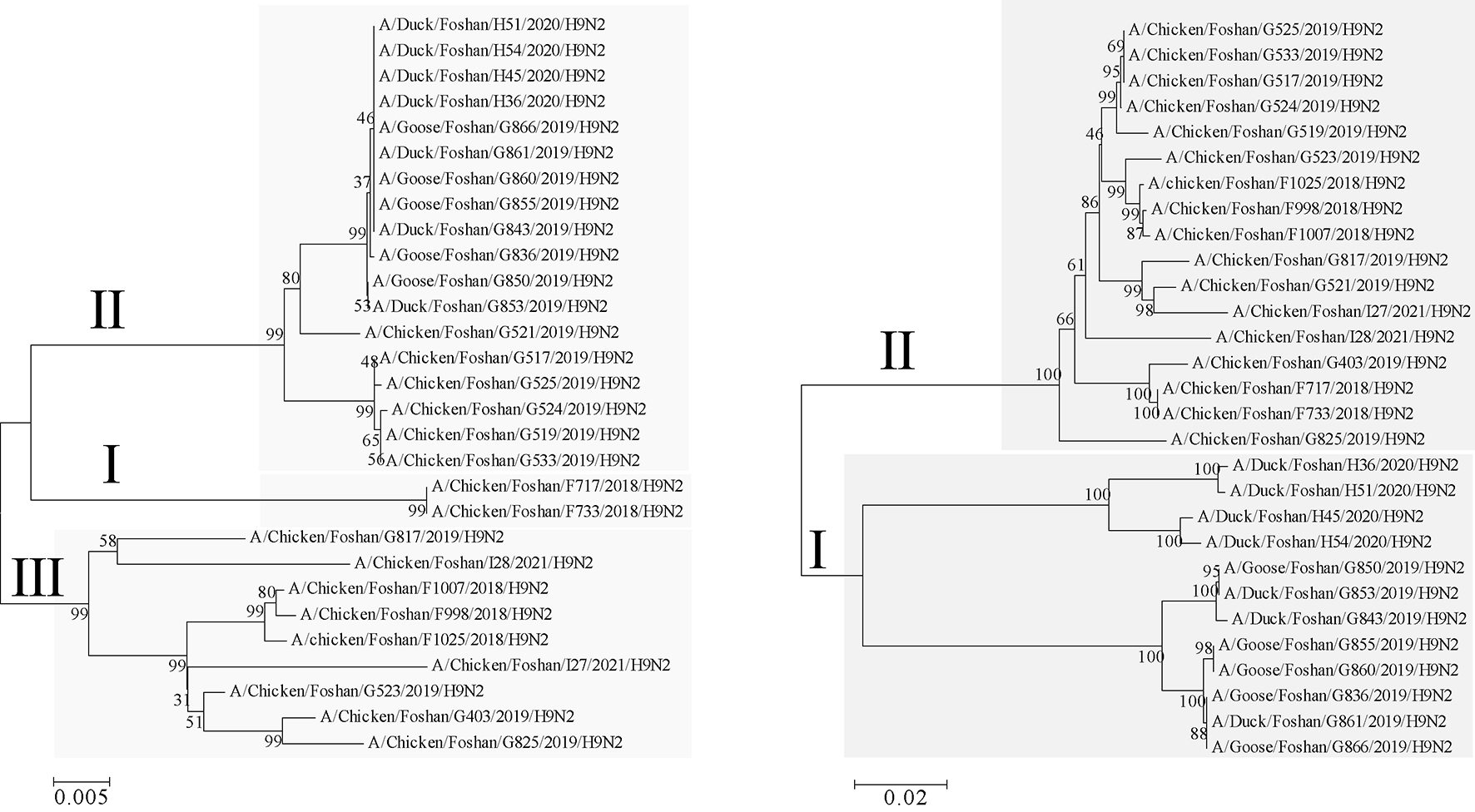

Supplement: Supplementary file 6 [file Image_1.TIF]
